# Development of a Reverse-Yield Factor Database Disaggregating Japanese Composite Foods into Raw Primary Commodity Ingredients Based on the Standard Tables of Food Composition in Japan

**DOI:** 10.3390/foods13070988

**Published:** 2024-03-24

**Authors:** Tatsuya Koyama, Kosuke Nakamura, Takashi Kiuchi, Shinji Chiba, Hiroshi Akiyama, Nobuo Yoshiike

**Affiliations:** 1 Graduate School of Health Sciences, Aomori University of Health and Welfare, 58-1 Mase, Hamadate, Aomori 030-8505, Aomori, Japan; koyama-ta@mimasaka.ac.jp (T.K.); n_yoshiike@ms.auhw.ac.jp (N.Y.); 2 Department of Food Science, Faculty of Human Life Sciences, Mimasaka University, 50 Kitazonocho, Tsuyama 708-8511, Okayama, Japan; 3 Division of Foods, National Institute of Health Sciences, 3-25-26 Tonomachi, Kawasaki-ku, Kawasaki 210-9501, Kanagawa, Japan; k-takashi@nihs.go.jp (T.K.); cshinji@nihs.go.jp (S.C.); h-akiyama@hoshi.ac.jp (H.A.); 4 Department of Analytical Chemistry, School of Pharmacy and Pharmaceutical Sciences, Hoshi University, 2-4-41 Ebara, Shinagawa-ku, Tokyo 142-8501, Japan

**Keywords:** food ingredient, composite food, exposure assessment, database, raw primary commodity, Japanese food

## Abstract

The reverse-yield factor (RF) database was developed for qualitatively and quantitatively disaggregating Japanese composite foods into raw primary commodity (RPC) ingredients. Representative equations for four types (dried, salted, fermented and mixed foods) were developed to calculate RFs using the food content and composition data for composite foods listed in the Standard Tables of Food Composition in Japan—2020—(STFCJ), published by the Ministry of Education, Culture, Sports, Science and Technology of Japan. Out of 1150 composite foods identified in the STFCJ, RFs for 54 dried, 41 salted, 40 fermented and 818 mixed foods were obtained. RFs for 197 mixed foods could not be calculated because these foods were produced from ingredients with no specified information and/or through complex processing. The content and composition of Japanese composite foods would be interpreted representatively by RFs in the developed database.

## 1. Introduction

Concerns have been raised regarding issues of food safety related to public health in Japan, in particular, exposure to hazardous chemicals, such as residual pesticides, veterinary drugs, feed additives, radioactive materials, toxic metals and other contaminants, through foods consumed by the Japanese population [1]. In general, this exposure is estimated based on food consumption data and the chemical composition of the respective foods, such as raw primary commodities (RPCs)—single-component foods that are unprocessed or whose nature is unchanged by processing (e.g., soybeans), RPC derivatives—single-component foods which have been physically changed by processing (e.g., soymilk) and composite foods (e.g., tofu) consisting of several components [2].

Food consumption data and chemical composition data have often been derived at different food levels. Food consumption data are normally obtained from dietary surveys on foods including composite foods [3] by food recording or recall methods for free-living individuals, while chemical compositions, especially residual pesticides, veterinary drugs and feed additives, have been predominantly determined through monitoring and testing on RPCs and RPC derivatives [4,5,6,7,8]. The RPCs and RPC derivatives consumption data are more suitable for the assessment of dietary exposure to those chemicals [2]. Therefore, definitions of composite foods in terms of their constituents and composition of food ingredients at the RPC and/or RPC derivative levels are indispensable in enabling a greater understanding of the total exposure to chemicals in the actual daily diet [9].

Reverse-yield factor (RF) is defined as the ratio of the amount of RPCs needed to obtain a given amount of an RPC-derived food product [2]. For example, according to a European Food Safety Authority (EFSA) report [2], RF for “apple juice” is reported as 1.54. This implies that 1.54 kg of fresh “apples” are needed to produce 1 kg of “apple juice”. When interpreting more complex RPC-derived food products back to their raw materials, a multi-tiered breakdown is required, with RFs of each processing step being multiplied to calculate the overall RF. Thus, each tier is considered, and RFs breaking down to the RPC are presented. For instance, “white rice” is processed through polishing from “brown rice” (RF 1.11), and “cooked rice” undergoes both polishing from “brown rice” and cooking in water (RF 0.38). A concept similar to RF, known as the diet correction factor (DCF), has been reported by the FAO/WHO Joint Meeting on Pesticide Residues (JMPR) [10]. DCF is used to avoid under-/over-estimation for assessing the acute dietary exposure to pesticide residues using the International Estimate of Short-Term Intake (IESTI) approach. For instance, the DCF from “cooked rice” to “white rice” was reported as 0.40. Differences between RF and DCF values may arise from variations in the processing information and its interpretation. The EFSA maintains a conversion table including RFs with references to each numerical value for breaking down RPC-derived food products back to the RPCs, with this information being available online [11].

Today, advanced methodologies are developed for more precisely estimating the risk from life-long consumer exposure to residues contaminating food commodities, such as pesticides, veterinary drugs and feed additives [12]. Various approaches for dietary exposure estimation are available depending on the purposes of the assessment and evaluation. Different methodologies, such as the total diet study, the duplicate diet method, the theoretical maximum daily intake (TMDI) and the estimated daily intake (EDI), are currently used for dietary exposure estimation in various jurisdictions and scientific bodies. For evaluating the exposure from the daily diet, a conservative approach for estimating the exposure at the RPC and/or RPC derivative levels would be useful. In Japan, all residual pesticides, veterinary drugs and feed additives are regulated to be below a uniform limit of 0.01 µg/g (called a default maximum residue limit [MRL]), except for pesticides that were set individual MRLs in foods, according to the positive list system officially established in 2006 by the Food Sanitation Act [13,14]. Those food items for which Japanese MRLs have been set according to the Japanese Food Sanitation Act [15] include RPCs and some RPC derivatives, single-component foods that have been physically changed by processing, such as pastes, juices, and oils, as well as livestock and fishery foods. The RPCs and RPC derivatives for which MRLs have been set need to be considered as potential sources of residues. The levels of exposure through the consumption of those foods on a long- and/or short-term basis may be estimated using the information on the amount of RPCs and RPC derivatives consumed and the amount of, for example, residual pesticides and veterinary drugs present, then compared with the Acceptable Daily Intake (ADI) and/or the Acute Reference Dose (ARfD). The ADI is that which, during an entire lifetime, poses no appreciable risk to the health of the consumer. The ARfD is an estimate of the amount of a substance in food and/or drinking water, normally expressed on a body weight basis, that can be ingested in a period of 24 h or less without any appreciable health risk to the consumer. These comparisons are made based on all known facts at the time of the evaluation of the chemical [16,17,18]. Consequently, the consumption data at the RPC and/or RPC derivative level are indispensable information for the dietary exposure estimation.

The exposure scenarios have been set for specific age groups according to individual food consumption data collected from the dietary survey. The EFSA has developed the RPC model by combining food consumption data with occurrence data including the residue concentration [19]. Food consumption data collected in the European Union (EU) were disaggregated into single-component foods that were then converted into unprocessed single-component foods using RF [11]. The RPC model is used by the Feed Additive Consumer Exposure FACE calculator to disaggregate individual food consumption data from the EU’s comprehensive database into the RPCs of animal origin, such as meat, eggs, and milk [2,12]. In Japan, the nationwide National Health and Nutrition Survey conducted by the Ministry of Health, Labour and Welfare of Japan has been used to obtain food consumption data that were specifically disaggregated into the Standard Tables of Food Composition in Japan (STFCJ)-registered food items, including composite foods [20,21,22]. Using RFs, the data on the composite foods that were consumed in Japan may be useful to representatively interpret the constituents and composition of food ingredients at the RPC and/or RPC derivatives level.

Composite foods are diverse; some are consumed worldwide, while others are often unique to a particular country or region. In Japan, more than 1000 different composite food items, including many traditionally unique to Japan, are officially registered in the STFCJ, a food composition table published and updated by the Ministry of Education, Culture, Sports, Science and Technology of Japan [23]. In the STFCJ—2020—(the Eighth Version) released in December 2020, 54 different sets of food composition data for 2478 food items, including dried, salted, fermented and other mixed foods, within which multiple ingredients are mixed, are registered. The food items registered in the STFCJ are regularly revised to take account of technological advancements, such as improved analytical methods for determining composition, and changes in circumstances involving foodstuffs, such as breeding or changes in cultivation methods [24,25]. The STFCJ thus officially presents the basic food items that are most representatively consumed by Japanese consumers, and therefore has been used as a reference for the annual nationwide National Health and Nutrition Survey and most dietary surveys for epidemiological studies.

RFs have not been comprehensively summarized for Japanese composite foods. The present study aims to calculate RFs for each composite food based on food content and composition data published in the STFCJ.

## 2. Materials and Methods

### 2.1. Categorization of Composite Foods

The information on the food items were referred to in the STFCJ—2020—(the Eighth Version) released in December 2020 [23]. The food items were divided into 18 different food groups: 1, Grains; 2, Potatoes and Starches; 3, Sugars and Sweeteners; 4, Pulses; 5, Nuts and Seeds; 6, Vegetables; 7, Fruit; 8, Mushrooms; 9, Algae; 10, Fish and Shellfish; 11, Meat; 12, Eggs; 13, Dairy Products; 14, Fats and Oils; 15, Confectionary; 16, Beverages; 17, Seasonings and Spices; and 18, Prepared and Processed Foods (Table 1). In the present study, the Prepared and Processed Foods group was not included in the analyses, because this food group has not been included in the nationwide National Health and Nutrition Survey in Japan. Composite foods were selected from the STFCJ, then categorized as dried, salted, fermented or mixed foods by registered Japanese dietitians (Tables S1 and S2).

### 2.2. RFs for Dried Foods

For dried foods, the water content and ingredient information in the STFCJ were used to calculate RFs. The weight of water (g), w, necessary to produce 100 g of dried food with a water content (%) of b, when processing an RPC with a water content (%) of a, can be written as Equation (1):

(1) $$\frac{a}{100}=\frac{b+w}{100+w}$$

Therefore, the weight of the RPC required to produce 100 g of dried food is shown by Equation (2).

(2) $$100+w=\frac{100\left(100-b\right)}{100-a}$$

Using Equations (1) and (2), RF for a dried food can theoretically be calculated using Equation (3).

(3) $${\mathrm{RF}}_{\mathrm{dried}\;\mathrm{food}}=\frac{100+w}{100}$$

### 2.3. RFs for Salted Foods

Salted foods processed using the RPCs and salt as the main ingredients were selected from the STFCJ. For these foods, information on their constituents and the composition of their food ingredients in the STFCJ was used to generate Equation (4).

(4) $$\frac{a}{100}=\frac{b+w}{100+w-s}$$

where a is the RPC with water content (%); w is the dehydrated weight of water (g); s is the added salt weight (g); and b is the resultant 100 g salted food water content (%).

The weight of the RPC needed to produce 100 g salted food can then be explained by Equation (5).

(5) $$100+w-s=\frac{100\left(100-s-b\right)}{100-a}$$

Using Equations (4) and (5), RFs for salted foods can be calculated using Equation (6).

(6) $${\mathrm{RF}}_{\mathrm{salted}\;\mathrm{foods}}=\frac{100+w-s}{100}$$

For the salted foods, vegetables pickled in salty rice bran paste, vinegar or soy sauce/miso were considered separately to calculate RFs. For vegetables pickled in salty rice bran paste, the main ingredients were considered to include vegetable, salt and rice bran. Thiamine in vegetables is considered to be negligible, while rice bran is considered to be rich in thiamine. RFs for vegetables pickled in salty rice bran paste were then calculated using the increased thiamine content data in the STFCJ by Equations (7) and (8),

(7) $${\mathrm{RF}}_{\mathrm{vegetables}}=\frac{100-s-b}{100-a}$$

(8) $${\mathrm{RF}}_{\mathrm{rice}\;\mathrm{bran}\;\mathrm{paste}}=\frac{c}{3.12}$$

where *a* is the RPC with water content (%); *s* is the added salt weight (%); *b* is the resultant 100 g salted food water content (%); *c* is the thiamine of vegetables pickled in salty rice bran (mg/100 g); and 3.12 is the thiamine content of rice bran paste (mg/100 g).

For vegetables pickled in vinegar, the main ingredients were taken to include vegetable, salt, vinegar and sugar. RFs for vegetables pickled in vinegar were calculated using the data on the increased carbohydrate content in the STFCJ, assuming that the increased acetate and sucrose in the composite food item were solely derived from vinegar and sugar and calculated using Equations (9)–(11).

(9) $${\mathrm{RF}}_{\mathrm{vegetables}}=\frac{100-s-b}{100-a}$$

(10) $${\mathrm{RF}}_{\mathrm{vinegar}}=\frac{d}{4.2}$$

(11) $${\mathrm{RF}}_{\mathrm{sugar}}=\frac{e-{\mathrm{RF}}_{\mathrm{vegetables}}\times f/100}{99.3}$$

where *a* is the RPC with water content (%); *s* is the added salt weight (%); *b* is the resultant 100 g salted food water content (%); *d* is the acetate content of vegetables pickled in vinegar (g/100 g); 4.2 is the acetate content of vinegar (%); *e* is the sucrose content of vegetables pickled in vinegar (%); *f* is the sucrose content of vegetables (%); and the sucrose content of sugar is taken as 99.3%.

For vegetables pickled in soy sauce/miso, the main ingredients were considered to be vegetables, salt, soy sauce/miso and sugar. In the present study, an increase in the weight of salt in the composite food item was assumed to originate from the increased amount of salt in the soy sauce/miso. Thus, RFs for vegetables pickled in soy sauce/miso were instead calculated based on the protein weight data in the STFCJ using Equations (12)–(16):

(12) $${\mathrm{RF}}_{\mathrm{vegetables}}=\frac{100-s-b}{100-a}$$

(13) $${\mathrm{RF}}_{\mathrm{soy}\;\mathrm{sauce}}=\frac{g}{7.7}$$

(14) $${\mathrm{RF}}_{\mathrm{miso}}=\frac{h}{12.5}$$

(15) $${\mathrm{RF}}_{\mathrm{sugar}}=\frac{\frac{i}{100}-{\mathrm{RF}}_{\mathrm{vegetables}}\times \frac{j}{100}-{\mathrm{RF}}_{\mathrm{soy}\;\mathrm{sauce}}\times \frac{0.1}{100}}{99.3}$$

(16) $${\mathrm{RF}}_{\mathrm{sugar}}=\frac{\frac{i}{100}-{\mathrm{RF}}_{\mathrm{vegetables}}\times \frac{j}{100}-{\mathrm{RF}}_{\mathrm{miso}}\times \frac{0}{100}}{99.3}$$

where *a* is the RPC with water content (%); *s* is the added salt weight (%); *b* is the resultant 100 g salted food water content (%); *g* is the protein content of vegetables pickled in soy sauce (%); 7.7 is the protein content of soy sauce (%); *h* is the protein content of vegetables pickled in miso (%); *I* is the sucrose content of vegetables pickled in soy sauce/miso (%); *j* is the sucrose of vegetables (%); and the sucrose content of sugar is taken as 99.3%.

Finally, RFs for vinegar and sugar as the ingredients were calculated by considering them as fermented foods, as described in the next section, and by subtracting RF for soy sauce/miso without sugar from that with sugar.

### 2.4. RFs for Fermented Foods

For fermented foods, food items that are processed and contain carbohydrates as the main constituent were selected. Of those selected, 34 different alcohol-fermented foods with information on the ethanol content in the STFCJ and 6 different acetic-acid-fermented foods with information on the acetic acid content in the STFCJ were classified as fermented foods (Table S2). RFs for the alcohol-fermented foods were calculated using Equation (17).

RF_alcohol-fermented food_ = (238 *a*/*c* + 100 *b*/*c*)/100 (17)

where *a* is the weight of alcohol (g/100 g) of the fermented food that has undergone alcoholic fermentation, *b* is the weight of available carbohydrates (g), and *c* is the weight of available carbohydrates per unit (g/100 g) of the raw material of the fermented food. The constant value “238” in Equation (17) was derived from the estimated amount of glucose in the raw material required to make ethanol in the alcohol fermentation.

RFs of the acetic-acid-fermented foods were calculated using Equation (18).

RF_acetic acid-fermented food_ = (238 *d*/*c* + 100 *b*/*c*)/100 (18)

where *d* is the weight of acetic acid (g/100 g) of the acetic-acid-fermented food, *b* is the weight of available carbohydrates (g), and *c* is the weight of carbohydrates (g/100 g).

### 2.5. RFs for Mixed Foods

For mixed foods, equations using the Gauss–Jordan elimination method [26] representing component values were established using the food composition values in the STFCJ as follows. Assuming that a composite food is made from *k* ingredients: C_1_, C_2_, …, C*_k_*, let *W_i_* be the weight of C*_i_* required to make 100 g of the composite food (*i* = 1, 2, …, *k*). The content and composition of nutrients in the composite food were taken from the STFCJ. The major nutrients that are considered to undergo no substantial changes during processing were selected then their values in the STFCJ were used to calculate RFs. With *P_j_* (*j* = 1, 2, …, *k*) denoting the weight of the nutrient contained in the composite foods, and *R_ij_* the weight of the nutrient contained in 100 g of C*_i_*, the following matrix equation can be constructed.

$$\frac{1}{100}\left[\begin{array}{cccc} R_{11} & R_{12} & \cdots & R_{1k}\\ R_{21} & R_{2k} & \cdots & R_{2k}\\ \vdots & \vdots & \ddots & \vdots \\ R_{k1} & R_{k2} & \cdots & R_{kk} \end{array}\right]\left[\begin{array}{c} W_{1}\\ W_{2}\\ \vdots \\ W_{k} \end{array}\right]=\left[\begin{array}{c} P_{1}\\ P_{2}\\ \vdots \\ P_{k} \end{array}\right]$$

Subsequently, *W_i_* is obtained from this matrix using the values (*R*, *P*) indicated in the STFCJ by the Gauss–Jordan elimination method. Consequently, the RF for the mixed foods was calculated by Equation (19).

RF _mixed foods_ = *W_i_*/100 (*i* = 1, 2, …, *k*) (19)

A graphical algorithm for developing the RF database is shown in Figure S1. The rationale for calculating the RF, equations used to calculate RFs for each composite food, and the RF database are available at the GitHub online, https://github.com/Reverseyieldfactor/Revese-yield-factors (accessed on 22 February 2024).

## 3. Results and Discussion

### 3.1. Definition of Food Types

**Figure 1:**
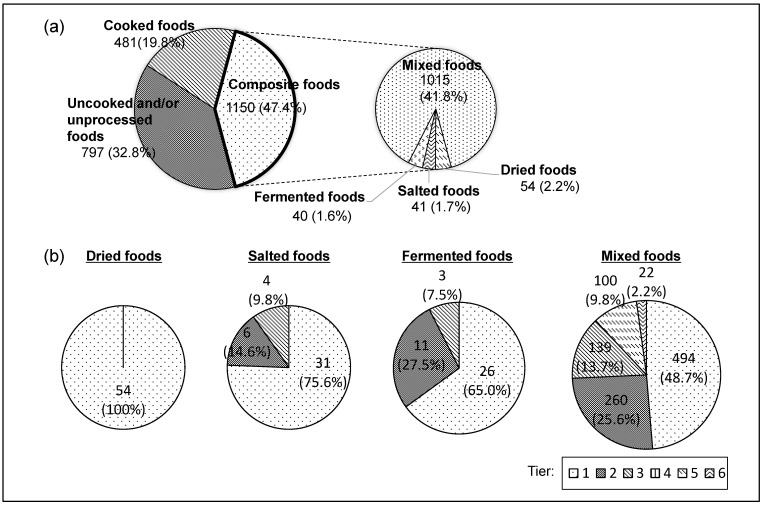
Food items in the STFCJ. (**a**) Food items were categorized into cooked, uncooked and/or unprocessed foods, and composite foods, with 1150 composite foods (47.4% of the total, except the prepared food items) being identified. (**b**) The “fermented”, “salted”, “dried” and “mixed” food items within the composite foods were disaggregated into food ingredients. The ratio of the number of steps required to disaggregate (tiers 1 to 6) is shown.

Of the 2478 food items listed in the STFCJ, 50 were in the Prepared and Processed Foods (food group 18), including those identified as pre-cooked and sold in markets, as reported previously [24,25]. To analyze the remaining 2428 food items for disaggregation into ingredients, the food items were first grouped into three categories: uncooked and/or unprocessed foods, cooked foods that include those home-cooked with a single component as an ingredient, and composite foods (Figure 1a). Those included 797 (32.8%) uncooked and/or unprocessed foods and 481 (19.8%) cooked foods. The cooking methods included boiling, cooking, steaming, microwave cooking and grilling, while non-thermal cooking methods included soaking in water and rehydration. The cooked foods produced with several ingredients including 45 types of food fried with oil or sautéed, 30 types of deep-fried foods (including fried, tempura, deep-fried, tonkatsu and fried chicken) and one type of glacé (candied food). As well as the uncooked and/or unprocessed and the cooked foods, 1150 (47.4%) composite foods were included and classified into 17 different food groups (Table 1). All 34 and 185 food items within the “fats and oils” and “confectioneries” groups, respectively, were identified as composite. Eleven food groups (“Grains”, “Sugars and Sweeteners”, “Pulses”, “Nuts and Seeds”, “Algae”, “Eggs”, “Dairy Products”, “Fats and Oils”, “Confectionaries”, “Beverages” and “Seasonings and Spices”) contained more composite food items than those in the uncooked/unprocessed and cooked groups. 

**Figure 2:**
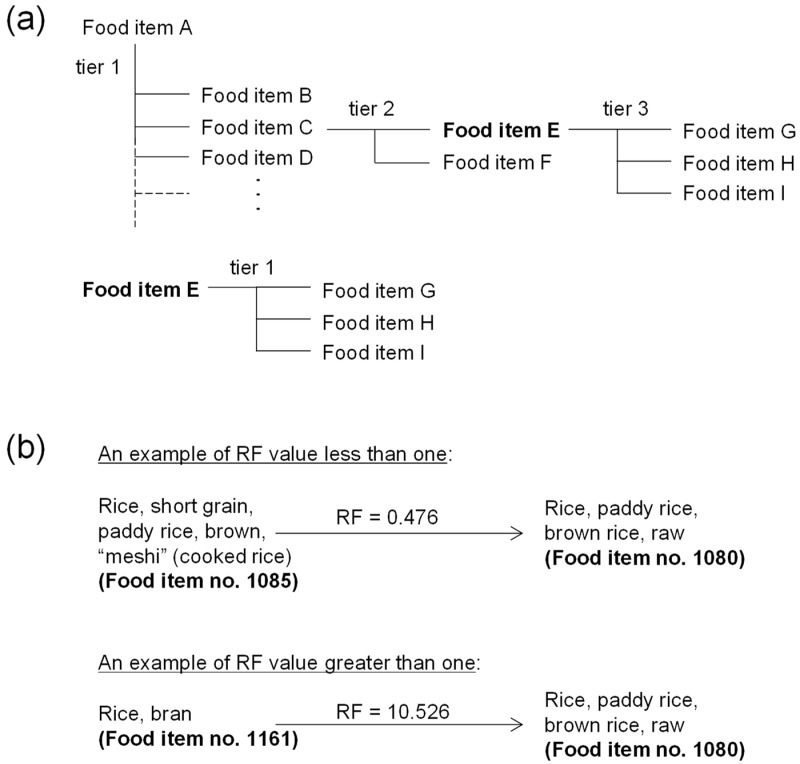
Disaggregation of composite food represented by RFs. (**a**) A dendrogram showing an example of a composite food (Food item A) that is disaggregated into food ingredients (Food items B–I) at tier 1–3. The composite food (Food item E) of an ingredient of Food item A, for example, is further disaggregated into its ingredients (Food items G–I) at tier 1. (**b**) An example of the food disaggregation into the RPC at an RF value less or greater than one. According to the food content and composition data in the STFCJ, Food item no. 1085 is produced by polishing the RPC (Food item no. 1080) and boiling with water, yielding an RF value of 0.476 (the RPC and water content at 47.6% and 52.4%, respectively). Food item no. 1161 is produced by polishing the RPC (Food item no. 1080), yielding RF value of 10.526.

Table 2 summarizes the numbers of the composite foods in the four different food types (dried, salted, fermented and mixed foods). Within the composite foods, a total of 54 (4.7%), 41 (3.6%) and 40 (3.5%) food items were grouped into dried, salted and fermented foods, respectively. Using the food composition data in the STFCJ, these composite foods were disassembled into food ingredients (Figure 2a). For mixed foods, 1015 (88.3%) food items within the composite foods group were included in the STFCJ, which contained information on the content ratio of 284 food items (24.7%) to directly calculate RF. Figure 2b shows that steamed or boiled rice (cooked rice, called “meshi” in Japanese, food item no. 1085), for example, can be explained by a single food ingredient, brown rice (food item no. 1080) with an RF value (0.476) of less than one, and rice bran (food item no. 1161) can similarly be explained by a single food ingredient, brown rice (food item no. 1080) with an RF value (10.526) of more than one. These calculated RF values of less than one indicated that the content of the food item as an ingredient after disassembly from a composite food was lower than that of the original composite food, and vice versa for those with a calculated RF value of more than one.

### 3.2. RFs for Dried Food Items

To calculate RFs for dried food items in the STFCJ, the water content data before and after the drying process was used. Using Equation (3), all 54 dried food items identified were disaggregated into the RPCs as ingredients at the first tier (the first disaggregation step of a composite food item into the RPC) by a single RF, meaning that no composite food as an ingredient was included in the item (Figure 1b).

### 3.3. RFs for Salted Food Items

To calculate the RFs for salted food items in the STFCJ, the water content data before and after food processing was used. The salted food items in the STFCJ were salty rice bran paste, vinegar, miso or soy sauce pickled foods, including those of traditional Japanese foods. The water content in the salt was assumed to be negligible, and the RFs for all the salted foods were calculated using Equation (6). There were a total of 31 (76%), 6 (15%) and 4 (10%) salted food items that were disaggregated into the RPCs at the tier 1, tier 2 and tier 3 of processing, respectively, using the calculated RFs (Figure 1b).

### 3.4. RFs for Fermented Food Items

To calculate RFs for fermented foods, the information on changes in food composition during processing presented in the STFCJ was used [27,28]. Various microorganisms are involved in fermentation, predominantly through the anaerobic pathway, with a complex set of chemicals including alcohol (i.e., ethanol), carbon dioxide and/or organic acids such as acetic, lactic and propionic acids. Fermented food items produced through fermentation with either alcohol, acetic acid or lactic acid were identified in the STFCJ, then 26 (65%), 11 (28%) and 3 (8%) food items were disaggregated into unprocessed single component foods at the tier 1, tier 2 and tier 3, respectively (Figure 1b). For the fermented food items, RFs were calculated at each tier. In the present study, composite foods that involved fermentation through more complex processing, such as nata de coco (fermented coconut water) and fermented crucian carp sushi, were not applicable to simple disassembly, so they were disregarded.

Alcohol-fermented Japanese composite foods are produced using microorganisms such as *Saccharomyces cerevisiae* (yeast) [29]. These food items include fermented alcoholic beverages, such as sake, beer and wine. Theoretically, through alcoholic fermentation, two molecules of ethanol and two molecules of carbon dioxide are produced from one molecule of glucose (C_6_H_12_O_6_ [glucose] → 2C_2_H_5_OH [ethanol] + 2CO_2_ [carbon dioxide]) but approximately 20% of the glucose is consumed for yeast growth during alcoholic fermentation, and 0.42 kg of ethanol is produced from 1 kg of glucose [30]. Therefore, 2.38 g (1/0.42 = 2.38) of glucose is required for 1 g ethanol in the composite food. Accordingly, the weight of the raw material was calculated using the glucose and carbohydrate contents of the raw material and the carbohydrate content of the fermented food. For example, the RPC to produce sake from rice (food item no. 16001 in the STFCJ) is polished rice. According to the STFCJ, the weights of ethanol and carbohydrate in sake are 12.3 and 2.5 g/100 g, respectively. The amount of carbohydrate is 83.1 g in 100 g of polished rice, so the weight (g) of polished rice required to make 100 g of sake is calculated to be 39.67 g (12.8 × 2.38 × 100/83.1 + 2.5 × 100/83.1 using Equation (17)). Therefore, the information in the STFCJ on the content of ethanol and carbohydrates in the fermented food and that of the available carbohydrates in the raw materials could be used to calculate RFs.

Japanese composite foods produced by acetic acid fermentation use microorganisms such as *Acetobacter* and *Komagataeibacter* [29]. Acetic acid (vinegar) is synthesized by metabolizing ethanol, thus the chemical reaction for acetic acid fermentation involves C_2_H_5_OH (ethanol) + O_2_ (oxygen) → CH_3_COOH (acetic acid) + H_2_O (water). Consequently, one molecule of ethanol and one molecule of oxygen produce one molecule of acetic acid and one molecule of water. Theoretically, 1.304 kg of acetic acid is produced from 1 kg of alcohol [30]. However, considering that the genus *Acetobacter* uses ethanol for its growth during fermentation so some ethanol may remain that has not been metabolized, approximately 1 kg of acetic acid was considered to be produced by metabolizing 1 kg of ethanol in the present study. As shown above, the weight of raw material glucose required to make 1 g of ethanol is 1/0.42 = 2.38 g. The weight of the raw material was then calculated using the weight information on the available carbohydrates in the raw material described in the STFCJ. Considering the molecular weights of glucose (approximately 180 g/mol), ethanol (approximately 46 g/mol) and acetic acid (approximately 60 g/mol), the amount of glucose derived from ethanol can be calculated as follows:

as *a*/46 × 1/2 × 180 = (45/23)*a* (g)

where *a* is the weight of ethanol (g) contained in the composite food.

The amount of glucose derived from acetic acid can be calculated as follows:

as *b*/60 × 1/2 × 180 = (3/2)*b* (g)

where *b* is the weight of acetic acid (g) contained in the composite food.

The food items in the STFCJ produced through acetic acid fermentation include black rice vinegar (food item no. 17090), grain vinegar (food item no. 17015), rice vinegar (food item no. 17016) and fruit vinegars (food item nos. 17017, 17018, 17091). Together, RFs for these acetic-acid-fermented foods were calculated from (238 *a*/*c* + 100 *b*/*c*)/100 using Equation (18).

Japanese composite foods produced by lactic acid fermentation include five food items (food item nos.: 13028, lactic acid bacteria beverages, not pasteurized after fermentation, milk solids non-fat content ≥ 3.0%; 13030, lactic acid bacteria beverages, milk solids non-fat content < 3.0%; 06070, cucumber, fruit, pickles, sour type (processed by lactic acid fermentation); 06115, Turnip, “sugukina”, leaves and root pickles; and 13029, lactic acid bacteria beverages, pasteurized after fermentation, milk solids non-fat content ≥ 3.0%). RFs for two food items (nos. 13028 and 13029) were calculated using carbohydrate and calcium content data in the STFCJ, while RFs for two food items (nos. 06070 and 06115) were calculated as salted foods. RF for food item no. 13030 could not be calculated due to the lack of ingredient information in the STFCJ.

### 3.5. RFs for Mixed Food Items

For the 731 mixed food items identified with no information on their content ratio in the STFCJ, insufficient information to directly calculate RFs was provided. To calculate RFs, an equation from the Gauss–Jordan elimination method was developed using the component values of the composite food and the RPCs in the STFCJ. It was assumed that the components of the total weight of the RPC used remained unchanged in the composite foods. For example, the RF of Japanese “udon” noodles (thick wheat noodles), which are representatively produced using two ingredients, flour and salt, could be established by the following method. As there are two unknown variables (the weights of flour and salt), two equations are needed to solve the problem. For the composite foods that have information on carbohydrate and salt used for ingredients as indicated in the STFCJ (Table S3), the following Equations (20) and (21) are used.

75.1 × *x*_flour_ + 0 × *x*_salt_ = 56.8/100 (20)

0 × *x*_flour_ + 99.5 × *x*_salt_ = 2.5/100 (21)

where x (g) is the weight of the individual ingredients per 100 g of udon.

The equations solved by the Gauss–Jordan elimination method lead to 494 (49%), 260 (26%), 139 (14%), 100 (10%) and 22 (2%) of the mixed foods being disaggregated into food ingredients at the RPC and/or RPC derivative levels in tier 1, tier 2, tier 3, tier 5 and tier 6 of processing, respectively, using the calculated RFs (Figure 1b).

### 3.6. Composite Foods for Which RFs Could Not Be Calculated

The RFs of 197 composite foods in the STFCJ could not be estimated due to a lack of information, the involvement of unknown or too many ingredients, or processing steps too complicated to quantitatively describe in terms of ingredients (Table 3). Table S4 shows that 94.9% of these composite foods (187 food items) contained unknown ingredients or had more ingredients than those given food content and composition data in the STFCJ. Therefore, the equations given earlier could not be used for calculating RFs, even by the Gauss–Jordan elimination method.

### 3.7. Limitations of RF Calculation for Composite Food Items

The processing of the RPCs may result in substantial differences in their nutrient contents. Therefore, a precise disaggregation of composite foods faces problems, such as: producers rarely declare the degree of processing of an ingredient on the package; the STFCJ provides only limited nutrient information on the RPCs and RPC derivatives; and many composite foods involve processing and/or cooking using different recipes. The present study proposes an approach for calculating RFs using the information on content and composition presented in the STFCJ. This approach may be used for composite foods with no information on the food label. However, the quality of ingredient composition calculations using our approach depends on several factors. First, the precise recognition and interpretation of the given ingredients, as well as a precise matching to the STFCJ entries, constitute a prerequisite. Second, the nutritional values provided in the food composition tables are average values representing a common example of an ingredient, which may vary because of natural influences and variations in the production process. Lastly, the component and nutritional values may change before and after processing, thus affecting the calculated RFs. Particularly for mixed foods, RFs calculated using the Gauss–Jordan elimination method may not well represent the component values before and after processing using the selected nutrient values in the STFCJ.

Regarding the method of calculating RF, a previous study reported the use of a computer program to analyze the ingredient composition [31]. As this method requires programming capabilities, it may not be sufficiently versatile and reproducible. Other studies [32,33,34] have described using mathematical equations to determine component values. However, these established methods require estimating the weight of raw materials and are based on the food composition tables and nutritional information within a particular country or region, and thus cannot simply be applied to the RPC conversion as the data available for Japanese composite foods are limited and need to be optimized. The present study, for the first time, proposes equations and concepts to calculate representative RFs for Japanese composite foods. In Japan, individual food consumption data from the national dietary surveys have been collected for food items registered in the STFCJ. These food items include not only the RPCs, but also their derivatives including composite foods. RFs set in the present study may best represent Japanese composite foods and be useful for obtaining a better estimate of the exposure to the chemical residues and a more precise risk assessment using the food consumption data collected in Japan. However, in light of the virtually endless unique sets of composite foods that may be produced with different and complex recipes, which may change over time, it is not advisable to set rigid RFs for individual composite foods.

This study summarized a snap-shot RF for Japanese composite foods at the time that this paper was prepared. Depending on the circumstances, RFs should be flexible; thus, using fixed values would not be appropriate. Subsequently, RFs for composite foods may differ depending on the specified components at the ingredient level, as well as on the algorithm. The STFCJ includes food composition tables and has been regularly revised, reflecting Japan’s gastronomic culture and changes in eating habits [24]. Comparison with the values that have been already reported, for example, by the EFSA and the JMPR, may provide further insights on the composite foods described in the STFCJ. According to the latest version of the STFCJ published, future work is necessary to determine and improve RFs calculated.

## 4. Conclusions

Japanese composite foods listed in the STFCJ were categorized into dried, salted, fermented and mixed foods to disaggregate into the RPCs and RPC derivatives. RFs for the composite foods in the STFCJ have been calculated and made available through a public database (https://github.com/Reverseyieldfactor/Revese-yield-factors [accessed on 22 February 2024]). The obtained RFs would be useful for understanding the RPCs and RPC derivatives likely to be contained at the ingredient level in the composite foods, and to provide an approach for assessing the exposure of Japanese consumers to hazardous chemicals through consumption of all foods, including composite foods.

## Figures and Tables

**Table 1:** Summary of food items listed in STFCJ.

Food Group No. and Name in STFCJ (Total Number of Food Items Listed)		Uncooked and/or Unprocessed Foods, Including Raw Agricultural Crops		Cooked Foods		Composite Foods	
		Number of Food Items	Percentage within Each Food Group (%)	Number of Food Items	Percentage within Each Food Group (%)	Number of Food Items	Percentage within Each Food Group (%)
1.	Grains (205)	12	5.9	60	29.3	133	64.9
2.	Potatoes and Starches (70)	16	22.9	28	40.0	26	37.1
3.	Sugars and Sweeteners (30)	2	6.7	0	0	28	93.3
4.	Pulses (108)	19	17.6	19	17.6	70	64.8
5.	Nuts and Seeds (46)	12	26.1	8	17.4	26	56.5
6.	Vegetables (401)	185	46.1	138	34.4	78	19.5
7.	Fruit (183)	101	55.2	2	1.1	80	43.7
8.	Mushrooms (55)	21	38.2	27	49.1	7	12.7
9.	Algae (57)	4	7.0	9	15.8	44	77.2
10.	Fish and Shellfish (453)	205	45.3	105	23.2	143	31.6
11.	Meat (310)	206	66.5	69	22.3	35	11.3
12.	Eggs (23)	5	21.7	7	30.4	11	47.8
13.	Dairy Products (59)	4	6.8	0	0	55	93.2
14.	Fats and Oils (34)	0	0	0	0	34	100
15.	Confectionaries (185)	0	0	0	0	185	100
16.	Beverages (61)	0	0	9	14.8	52	85.2
17.	Seasonings and Spices (148)	5	3.4	0	0	143	96.6
	Subtotal (2428)	797	32.8	481	19.8	1150	47.4
18.	Prepared and Processed Foods (50)						
	Total (2478)						

**Table 2:** Composite food items (*n* = 1013) listed in STFCJ.

Food Group No. and Name in STFCJ (Total Number of Composite Food Items Listed)		Dried Food		Salted Food		Fermented food		Mixed food			
		Number of Food Items	Percentage within Each Food Group (%)	Number of Food Items	Percentage within Each Food Group (%)	Number of Food Items	Percentage within Each Food Group (%)	Number of Food Items with No Information on Content Ratio	Percentage within Each Food Group (%)	Number of Food Items with Information on Content Ratio for RF Calculation	Percentage within Each Food Group (%)
1.	Grains (133)	0	0	0	0	0	0	92	69.2	41	30.8
2.	Potatoes and Starches (26)	0	0	0	0	0	0	24	92.3	2	7.7
3.	Sugars and Sweeteners (28)	0	0	0	0	0	0	27	96.4	1	3.6
4.	Pulses (70)	2	2.9	0	0	0	0	61	87.1	7	10.0
5.	Nuts and Seeds (26)	0	0	0	0	0	0	26	100	0	0
6.	Vegetables (78)	7	9.0	28	35.9	0	0	37	47.4	6	7.7
7.	Fruit (80)	6	7.5	3	3.8	0	0	71	88.8	0	0
8.	Mushrooms (7)	3	42.9	0	0	0	0	4	57.1	0	0
9.	Algae (44)	5	11.4	0	0	0	0	39	88.6	0	0
10.	Fish and Shellfish (143)	26	18.2	10	7.0	0	0	107	74.8	0	0
11.	Meat (35)	0	0	0	0	0	0	35	100	0	0
12.	Eggs (11)	3	27.3	0	0	0	0	3	27.3	5	45.5
13.	Dairy Products (55)	2	3.6	0	0	0	0	50	90.9	3	5.5
14.	Fats and Oils (34)	0	0	0	0	0	0	34	100	0	0
15.	Confectionaries (185)	0	0	0	0	0	0	18	9.7	167	90.3
16.	Beverages (52)	0	0	0	0	34	65.4	18	34.6	0	0
17.	Seasonings and Spices (143)	0	0	0	0	6	4.2	85	59.4	52	36.4
	Total (1150)	54	4.7	41	3.6	40	3.5	731	63.6	284	24.7

**Table 3:** Reasons why RF values could not be calculated.

Food Group No. and Name in STFCJ (Total Number of Applicable Food Items)		Ingredients Unknown		Too Many Ingredients		Complicated Processing	
		Number of Food Items	Percentage within Each Food Group (%)	Number of Food Items	Percentage within Each Food Group (%)	Number of Food Items	Percentage within Each Food Group (%)
1.	Grains (4)	4	100	0	0	0	0
2.	Potatoes and Starches (6)	6	100	0	0	0	0
3.	Sugars and Sweeteners (27)	27	100	0	0	0	0
4.	Pulses (0)						
5.	Nuts and Seeds (17)	17	100	0	0	0	0
6.	Vegetables (5)	4	80	1	20	0	0
7.	Fruit (7)	6	86	0	0	1	14
8.	Mushrooms (2)	2	100	0	0	0	0
9.	Algae (26)	26	100	0	0	0	0
10.	Fish and Shellfish (15)	14	93	0	0	1	7
11.	Meat (3)	3	100	0	0	0	0
12.	Eggs (0)						
13.	Dairy Products (4)	4	100	0	0	0	0
14.	Fats and Oils (13)	13	100	0	0	0	0
15.	Confectionaries (2)	2	100	0	0	0	0
16.	Beverages (24)	24	100	0	0	0	0
17.	Seasonings and Spices (42)	35	83	7	17	0	0
	Total (197)	187	95	8	4	2	1

## Data Availability

The original contributions presented in the study are included in the article/Supplementary Materials, further inquiries can be directed to the corresponding author.

## Supplementary Materials

The following supporting information can be downloaded at https://github.com/Reverseyieldfactor/Revese-yield-factors (accessed on 22 February 2024): rationale for calculating RF, equations used to calculate RFs for each composite food, and RF database. The following supporting information can be downloaded at: https://www.mdpi.com/article/10.3390/foods13070988/s1, Figure S1: Graphical algorithm for developing RF database using the information in STFCJ; Table S1: Food type of food items in STFCJ; Table S2. Composite food type of food items in STFCJ; Table S3: Composition of Japanese “udon” noodles (thick wheat noodles) and content information in STFCJ; Table S4: List of Japanese composite food items in STFCJ whose RF values could not be calculated.
