# Supplementary material for: Development of a Reverse-Yield Factor Database Disaggregating Japanese Composite Foods into Raw Primary Commodity Ingredients Based on the Standard Tables of Food Composition in Japan
Source: Foods. 2024 Mar 24;13(7):988. doi: 10.3390/foods13070988 (PMC11011496; doi:10.3390/foods13070988)
Supplement: Supplementary file 1 [file foods-13-00988-s001.zip › Figure S1.pptx]

## Slide 1
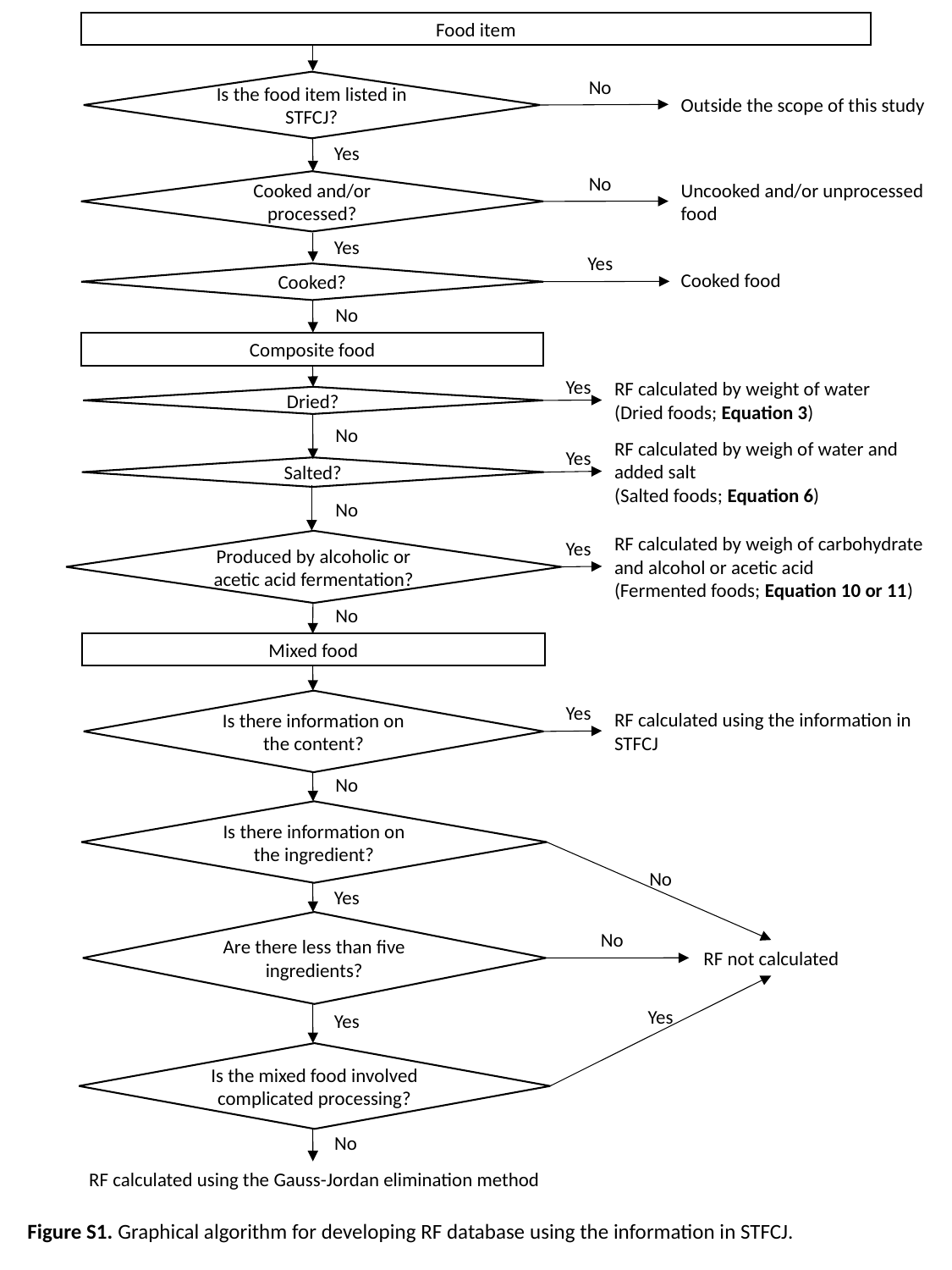

Food item
No
Is the food item listed in STFCJ?
Outside the scope of this study
Yes
No
Cooked and/or processed?
Uncooked and/or unprocessed food
Yes
Yes
Cooked food
Cooked?
No
Composite food
Yes
RF calculated by weight of water
(Dried foods; Equation 3)
Dried?
No
RF calculated by weigh of water and added salt
(Salted foods; Equation 6)
Yes
Salted?
No
RF calculated by weigh of carbohydrate and alcohol or acetic acid
(Fermented foods; Equation 10 or 11)
Yes
Produced by alcoholic or acetic acid fermentation?
No
Mixed food
Is there information on the content?
Yes
RF calculated using the information in STFCJ
No
Is there information on the ingredient?
No
Yes
Are there less than five ingredients?
No
RF not calculated
Yes
Yes
Is the mixed food involved complicated processing?
No
RF calculated using the Gauss-Jordan elimination method
Figure S1. Graphical algorithm for developing RF database using the information in STFCJ.
